# Eccrine Carcinoma With an Unknown Primary: Managing Occult Cancer Through Multidisciplinary Tumor Board Collaboration

**DOI:** 10.7759/cureus.23183

**Published:** 2022-03-15

**Authors:** Melissa Mariano, Chinmay Jani, Prateek Khanna, Dipesh Patel, John Perry, Bhargavi Yalamarti, Anthony Abner

**Affiliations:** 1 Internal Medicine, Mount Auburn Hospital, Harvard Medical School, Cambridge, USA; 2 Radiology, Mount Auburn Hospital, Harvard Medical School, Cambridge, USA; 3 Pathology, Mount Auburn Hospital, Harvard Medical School, Cambridge, USA; 4 Hematology and Oncology, Mount Auburn Hospital, Harvard Medical School, Cambridge, USA; 5 Radiation Oncology, Mount Auburn Hospital, Harvard Medical School, Cambridge, USA

**Keywords:** radiation, lymphadenopathy, unknown primary, primary mucinous carcinoma, eccrine carcinoma

## Abstract

Eccrine carcinomas are rare cutaneous cancers that tend to be locally aggressive. Here we report a rare case of a mucinous eccrine carcinoma presenting in axillary lymph nodes without an identifiable primary lesion. This is a 69-year-old male with a past medical history of benign prostatic hyperplasia, melanoma, basal cell carcinoma, hypercholesterolemia, hypertension, and arthritis who was found to have an elevated prostate-specific antigen. Transrectal prostate biopsies confirmed adenocarcinoma of the prostate. A chest CT scan performed for further staging of prostate cancer identified new left axillary lymphadenopathy and positron emission tomography (PET)-CT imaging showed moderate fluorodeoxyglucose (FDG) uptake in the lymph nodes of the left axilla and left subpectoral regions. Lymph node tissue obtained by core needle biopsy demonstrated high-grade carcinoma with a nonspecific immunohistochemical profile. Complete left axillary lymphadenectomy was performed, revealing mucinous eccrine carcinoma. He was started on hormonal therapy for prostate cancer and radiation therapy for axillary eccrine carcinoma at the same time. Based on our literature review, this appears to be the first case of eccrine carcinoma in axillary lymph nodes with an unknown primary. This case is further complicated by synchronous primary prostate cancer. After a multidisciplinary tumor board review, it was decided that his axillary disease should be treated as a primary mucinous carcinoma with complete lymphadenectomy followed by localized radiation. The patient had stable disease at the six-month follow-up. Cancers with unknown primary lesions pose unique challenges in disease management. Without established recommendations or guidelines, multidisciplinary discussions and a collaborative approach are needed.

## Introduction

Skin cancer is the most commonly diagnosed type of cancer [1]. The primary types of skin cancer include basal cell and squamous cell carcinomas and, less commonly, melanoma, which has worse outcomes. Cutaneous adnexal tumors are rare skin cancers typically classified by the skin structures from which they arise, including hair follicles, sebaceous glands, apocrine glands, and eccrine glands. Most cutaneous adnexal tumors demonstrate apocrine or eccrine differentiation and can be divided into benign or malignant tumors [2]. Eccrine carcinomas represent less than 0.01% of diagnosed cutaneous malignancies [3]. These sweat gland carcinomas may be further classified by type: porocarcinomas, syringomatous carcinomas, ductal carcinomas, adenoid cystic carcinomas, and mucinous carcinomas [4]. Primary cutaneous mucinous carcinomas typically present in the seventh decade of life and have a higher incidence in women [5,6]. The head and neck (particularly the eyelids and scalp) are the most common locations for these primary lesions [5,6]. Histologic diagnosis of primary cutaneous adnexal tumors may be difficult due to significant overlap in histopathologic and immunohistochemical profiles, warranting a thorough history and diagnostic imaging studies [5]. As primary cutaneous malignancies tend to be solitary, locally aggressive lesions with limited ability to metastasize, surgery or localized radiation are often the preferred treatment [7]. There have been reports of attempted treatment with targeted therapies or adjuvant chemotherapy for patients with metastases [8-11]. Here we report a rare case of a mucinous carcinoma presenting in axillary lymph nodes with no known primary lesion.

## Case presentation

A 69-year-old male with a past medical history of benign prostatic hyperplasia, melanoma post-Mohs surgery on his back, basal cell carcinoma post-Mohs surgery on his forehead and back, restless leg syndrome, hypercholesterolemia, hypertension, and arthritis presented to his primary care physician for a Medicare annual wellness exam complaining of intermittent pelvic pain. He denied any smoking history and endorsed drinking two six-packs of beer on weekends. He had no significant family history or occupational history. His melanoma was removed 15 years prior, and he has since been followed regularly by a dermatologist. On physical exam, his abdomen was soft and non-distended without masses, organomegaly, or hernias. Some mild tenderness was noted in the right lower quadrant and normoactive bowel sounds were auscultated. A rectal examination was not performed. Scars from previous melanoma removal were visualized, but no new lesions were identified. His prostate-specific antigen (PSA) was found to be elevated (13.0 ng/mL) compared with a previous PSA drawn three years prior (4.1 ng/mL) with no other interval measurement. A confirmatory PSA post-antibiotic administration was 17.2 ng/mL.

**Figure 1:**
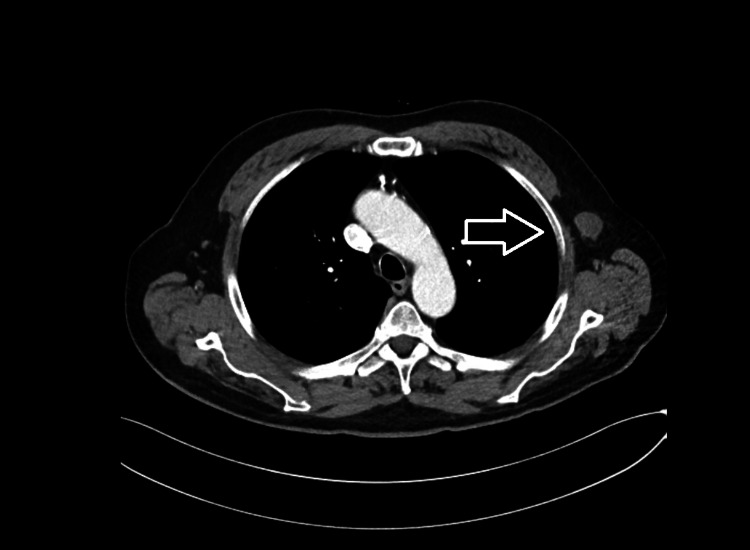
Axial view of chest CT scan with contrast demonstrating multiple left axillary and subpectoral lymph nodes, the largest of which measured 3.3 x 1.9 cm (white arrow).

**Figure 2:**
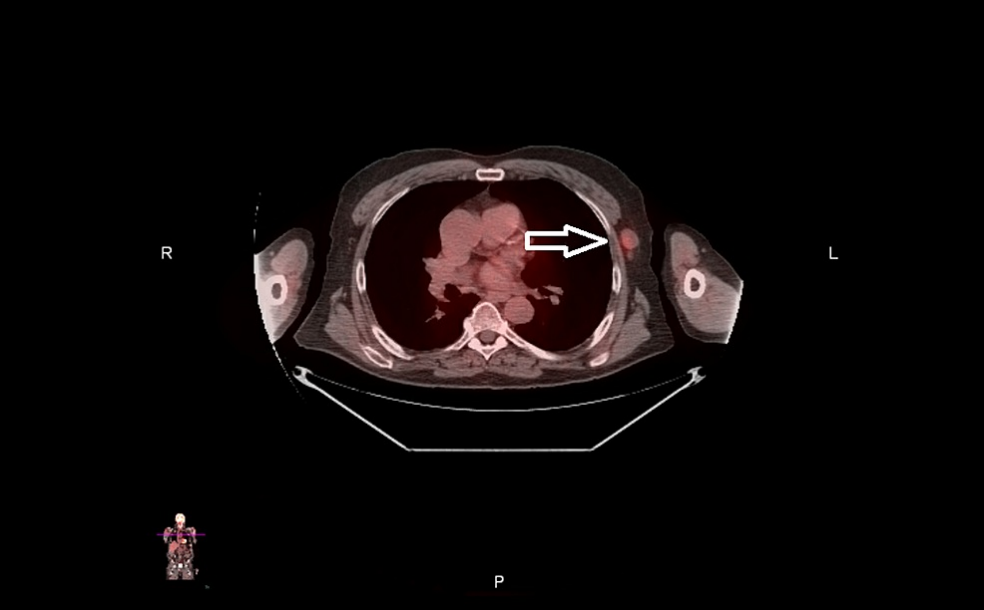
Axial view of PET-CT scan showing moderate FDG uptake in the lymph node of the left axillary/subpectoral region (white arrow). PET: positron emission tomography; FDG: fluorodeoxyglucose

He underwent transrectal prostate biopsies, which were positive for adenocarcinoma. Pathology revealed a Gleason score of 9 (4+5) present in two cores from the right base, with an additional core of Gleason 8 (4+4) disease from the left base. Subsequent staging CT imaging of the abdomen pelvis incidentally identified a solitary 6.5 mm nodule in the lungs, but no axillary, mesenteric or retroperitoneal lymphadenopathy, or bone metastases. In the interim, leuprolide hormonal therapy was initiated for his prostate cancer. Five weeks later, a follow-up CT scan of the chest showed near-complete resolution of the right lower lobe 6.5 mm pulmonary nodule, which was attributed to an infectious or non-inflammatory process. Additionally, there was a new 6 mm pulmonary nodule in the superior segment of the right lower lobe and a smaller pulmonary nodule in the left upper lobe, as well as new left axillary lymphadenopathy (Figure 1). Further imaging with positron emission tomography (PET)-CT scan was performed, which showed moderate fluorodeoxyglucose (FDG) uptake in the lymph nodes of the left axilla and left subpectoral regions (Figure 2). No other evidence of FDG avidity was noted on the PET-CT scan, and no lesions were identified on a bone scan.

Given the new finding of lymphadenopathy, a core needle biopsy of an axillary lymph node was performed, which demonstrated morphologic features and positive staining for epithelial markers. Specifically, sections of the biopsy showed malignant epithelial cells with enlarged, atypical nuclei and minimal cytoplasm, predominantly forming cohesive clusters on a background of extracellular, pale blue mucin, infiltrating fibromuscular stroma. A broad panel of immunohistochemical stains was performed. The neoplastic cells were positive for keratin AE1/AE3, keratin 7, GATA3 (seen in breast, urothelial, and salivary gland primary tumors), and p63 (seen in squamous tumors of any origin or urothelial primary tumors). The neoplastic cells were negative for keratin 20, PSA, prostate-specific acid phosphatase (PSAP), thyroid transcription factor 1 (TTF-1), CDX2, synaptophysin, mammaglobin, gross cystic disease fluid protein 15 (GCDFP-15), estrogen receptor (ER), progesterone receptor (PR), uroplakin, and S100. Napsin A showed faint blush staining that was considered to be equivocal. Human epidermal growth factor receptor 2 (HER2) showed faint staining (score 1-2+). A mucin stain highlighted faint background extracellular mucin. Additional stains showed that the neoplastic cells were positive for keratin 5/6. P16 was negative for block positivity (only rare, weak staining was present). SOX10 and PAX8 were negative. These results suggested a high-grade carcinoma with a nonspecific immune profile for the site of origin. 

**Figure 3:**
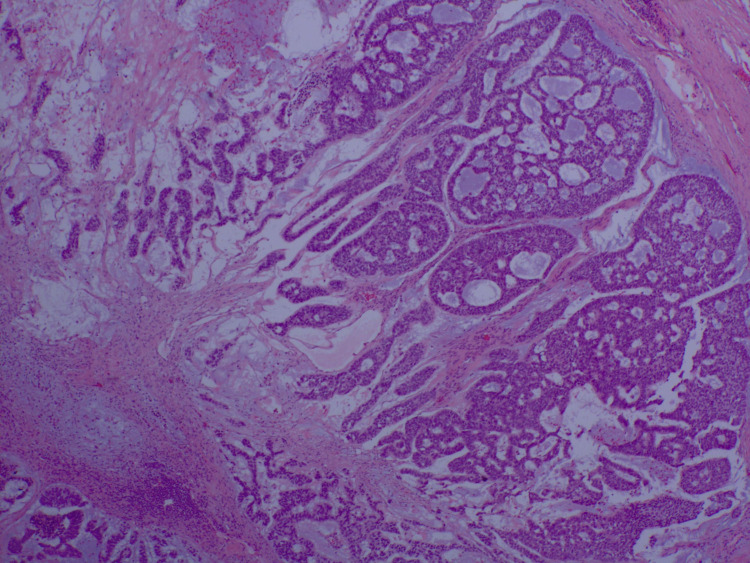
H&E section showing basaloid cells infiltrating tubules and glands with 40x magnification. The specimen demonstrates lobulated lesions divided by fibrous septa. H&E: hematoxylin and eosin stain

**Figure 4:**
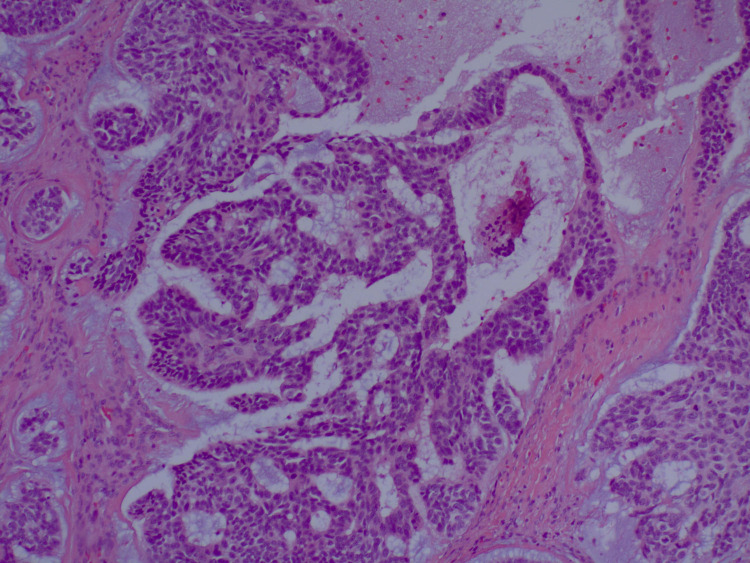
H&E section of higher magnification illustrating basaloid nature of malignant cells (60x magnification). Within each lobule, there are islands and trabeculae of relatively monomorphic cells having rounded or ovoid nuclei and limited amounts of palely eosinophilic cytoplasm. The cells are distributed within a prominent mucinous matrix. H&E: hematoxylin and eosin stain

He then underwent complete left axillary lymphadenectomy and was found to have seven of 13 lymph nodes positive for malignancy. The specimen showed a lobulated lesion in which the lobules were divided by fibrous septa (Figure 3). Within each lobule, there were islands and trabeculae of relatively monomorphic cells having rounded or ovoid nuclei and limited amounts of palely eosinophilic cytoplasm. The cells were distributed within a very prominent mucinous matrix and scattered mitotic figures were observed (Figure 4). Lesional cells were positive for cytokeratin AE1/AE3, GATA, p40, p63, cytokeratin 5/6, cytokeratin 7, and beta-catenin. Patchy staining for neuron-specific enolase (NSE) and CD56 was noted. Lesional cells were negative for ER, PR, Her2, S100, smooth muscle antibody (SMA), CD31, CD34, chromogranin, CK19, D2-40, desmin, epithelial membrane antigen (EMA), GCDFP, mammaglobin, Napsin, PAX8, PSAP, PSA, synaptophysin, thyroglobulin, TTF1, uroplakin, CDX2, and cytokeratin 20. The morphology of the tumor was found to be unusual and the immunophenotype was not entirely specific, thus the specimen was sent to a local high-volume academic center for consultation and further review. The final pathology report stated that the immunohistochemical stains demonstrated diffuse positivity for pan-keratin and p63, as well as focal positivity for EMA in the ducts, all of which are epithelial markers. Stains for S100 protein (marker for melanoma or nerve sheath tumors), CEA (nonspecific marker for colorectal, pancreatic, gastric, breast, or thyroid cancers), and PLAG1 (pleomorphic adenoma) were negative, while there was equivocal positivity for MYB (proto-oncogene for leukemia), which was confirmed to be negative with fluorescence in situ hybridization (FISH) testing. These histologic features and immunochemical stains were found to be most consistent with mucinous eccrine carcinoma.

His case was discussed at the multidisciplinary tumor board multiple times as a primary prostate adenocarcinoma with concurrent mucinous eccrine carcinoma of unknown origin. It was determined that for the mucinous carcinoma, he should undergo radiation therapy after surgical excision (axillary lymph node dissection). For the prostate adenocarcinoma, it was recommended that he complete definitive radiation therapy with combined androgen deprivation therapy for 18 months due to his high-grade disease. He had stable disease at the six-month follow-up.

## Discussion

Based on our literature review, this is the first case of eccrine carcinoma with an occult primary. The differential diagnosis for this case was complicated by an active, synchronous prostate adenocarcinoma and pulmonary nodules. Axillary lymphadenopathy was incidentally identified on staging CT imaging for the prostate malignancy. Initially, the differential diagnosis for the enlarged lymph nodes included metastasis from prostate cancer, primary or metastatic cancer of pulmonary origin, or reactive lymphadenopathy. The lung nodules identified on CT imaging appeared and resolved within four months, which is rapid for metastatic or primary lung cancer, indicating more likely a non-inflammatory infectious process. Fine needle aspiration biopsy of the axillary lymph node confirmed high-grade carcinoma based on the morphologic features and positive staining for epithelial markers (keratin AE1/AE3, keratin 5/6, keratin 7), GATA 3, and p63. However, the immunohistochemical profile was nonspecific for the site of origin. Notably, prostate markers (PSA, PSAP) and melanoma markers (S100) were negative.

At this point, the differential included skin squamous cell carcinoma, primary lung cancer, metastatic breast cancer, or head and neck primary. PET-CT and bone scans did not identify a second primary source, thus minimizing the likelihood of metastasis from the lungs or elsewhere. It was also less likely to be a distant metastasis from his primary prostate adenocarcinoma given the positive epithelial markers and negative prostate markers (PSA, PSAP) on core biopsy, as well as the lack of lymphadenopathy in the abdomen or pelvis. For treatment, he underwent complete axillary lymphadenectomy. Final pathology revealed positive pan-keratin staining, p63, and EMA focally in the ducts, while S100, pleiomorphic adenoma gene 1 (PLAG1), CEA, and MYB were negative. After reviewing the results with multiple colleagues including dermatopathologists, the reading pathologist determined that the data appeared most consistent with mucinous eccrine carcinoma in the axillary lymph nodes. Still, there was no primary cutaneous lesion identified.

Carcinomas with an unknown primary are difficult to manage. There is limited information surrounding the diagnosis and prognosis of these malignancies; therefore, they require a broad multidisciplinary approach. Cancer of unknown origin is defined as a metastatic tumor for which no primary anatomical site is identified following a comprehensive clinical evaluation [12]. The site of clinical presentation dictates management for cancers with unknown primaries. Thus, when the primary site is unknown, first-line therapy choices may be guided by clinical and pathological features such as immunohistochemical stains or molecular gene expression profiling [12]. Current National Comprehensive Cancer Network® (NCCN®) guidelines for occult primaries recommend a thorough initial evaluation, including a complete history and physical, comprehensive lab work, and pan-CT imaging or endoscopy (as indicated) [13]. Further workup involves a biopsy of the most accessible site (core needle biopsy preferred) with immunohistochemical stains, tumor mutational burden determination, and consideration for further genetic testing. The pathological diagnosis guides the next steps for evaluation.

Although cancer treatments tend to be directed by anatomical site, in some instances different types of cancer may present in the same location with remarkably similar histology. For instance, skin lesions may be primary skin tumors or metastases from other sites. Breast cancer is the most common source for cutaneous metastases and appears remarkably similar to eccrine carcinoma under the microscope. Breast tissue is composed of modified sweat glands and, hence, it may be nearly impossible to distinguish the two malignancies on histology alone [14]. Prognosis and treatment differ significantly between eccrine and breast cancers; therefore, it is essential that all data points be considered when determining the correct diagnosis. A multidisciplinary tumor board discussed his case at length in collaboration with local high-volume academic centers to determine the most appropriate course of action for his case. Although the primary cutaneous anatomical site remained unidentified, it was decided that he should be treated for primary mucinous eccrine carcinoma and primary prostate cancer simultaneously.

Cutaneous mucinous carcinoma is a low-grade, slow-growing neoplasm with weak metastatic potential [5]. Lesions tend to present as elevated, painless, erythematous nodules with crusting or ulceration that vary greatly in size [5,6]. Primary mucinous carcinoma is often misdiagnosed as a cutaneous metastasis, both clinically and histologically [5]. Mucinous eccrine carcinoma may be very difficult to distinguish from other types of metastatic disease including secondary breast, lung, prostate, ovarian, or gastrointestinal neoplasms. Mucinous eccrine tumor cells present as cuboidal or columnar cells that are “typically arranged in nests and clusters with the mucin-filled cavities separated by fibrous strands” and appear to have low mitotic count and little nuclear atypia [5]. They tend to be +CK7, -CK20, which makes them immunohistochemically similar to breast and lung cancers, but the -CK20 marker differentiates them from gastrointestinal cancers (+CK20) [5]. In general, malignant cutaneous adnexal tumors such as eccrine carcinomas tend not to metastasize, but nodal disease and distant metastases may occur in approximately 7% and 3-12% of cases, respectively [7,15,16]. Patients with metastatic eccrine carcinoma have rapid disease progression with increased morbidity and mortality. There is also a high risk for recurrence and poor treatment response of recurrent disease [5,6,15]. Younger age, location of tumors on the trunk, and larger initial tumors are worse prognostic factors [6]. Mean overall survival is approximately 11 years with 85% survival after five years and 87% survival after 10 years [5].

Management for primary eccrine carcinoma typically involves wide local excision with or without lymph node dissection. Due to the rarity of lymph node involvement, there is insufficient data to recommend routine lymph node biopsy in patients without clinical lymph node involvement [16]. There does not appear to be a survival benefit in patients receiving adjuvant radiation therapy versus surgery alone [17]. Some cases of metastatic eccrine carcinomas have been treated with chemotherapy, but there is minimal evidence from only a few clinical reports [18]. One case study reports successful treatment of metastatic eccrine tumors expressing estrogen and progesterone receptors with hormone-directed therapies. In contrast, another case study demonstrated an excellent response to treating an HER-2 expressing tumor with trastuzumab [8,11,19]. In the absence of data and randomized clinical trials, the treatment for eccrine carcinoma must be tailored to each patient's circumstances.

## Conclusions

Typical oncologic disease management depends on the primary lesion site. If the primary site is unknown, comprehensive investigations including a detailed history and physical, broad laboratory workup, complete CT imaging, and immunohistochemical staining of tissue samples should be performed to narrow the differential diagnoses. After differential diagnoses have been ruled out, management options can be explored based on primary lesion site of the most likely diagnosis. For this patient's prostate adenocarcinoma, his treatment plan was decided through consultation of evidenced-based guidelines. With regards to his rare mucinous eccrine carcinoma metastasis without a primary lesion, treatment planning required thorough review of the literature and collaborative multidisciplinary discussions. When the evidence base for particular cancers is sparse, providers must rely on their own clinical judgement, consultation of the existing literature, and collaboration with colleagues to provide the best care for patients with rare diagnoses.
